# d-(+)-Galactose-Conjugated Single-Walled Carbon Nanotubes as New Chemical Probes for Electrochemical Biosensors for the Cancer Marker Galectin-3

**DOI:** 10.3390/ijms12052946

**Published:** 2011-05-05

**Authors:** Young Kum Park, Bayarmaa Bold, Woo Kyung Lee, Min Hyon Jeon, Kay Hyeok An, Seung Yol Jeong, Young Key Shim

**Affiliations:** 1 Department of Nano System Engineering, Inje University, Gimhae, 612-749, Korea; E-Mails: immergreen@dreamwiz.com (Y.K.P.); bayarma_sbmt@yahoo.com (B.B.); wlee@inje.ac.kr (W.K.L.); mjeon@inje.ac.kr (M.H.J.); 2 Carbon Valley R & D Division, Jeonju Institute of Machinery and Carbon Composites, Jeonju, 561-844, Korea; E-Mail: khan@jmc.re.kr; 3 Nano Carbon Materials Research Group, Korea Electrotechnology Research Institute, Changwon, 641-120, Korea; E-Mail: syjeong@keri.re.kr

**Keywords:** biosensor, cancer marker, galectin-3, galactose, single-walled carbon nanotubes

## Abstract

d-(+)-Galactose-conjugated single-walled carbon nanotubes (SWCNTs) were synthesized for use as biosensors to detect the cancer marker galectin-3. To investigate the binding of galectin-3 to the d-(+)-galactose-conjugated SWCNTs, an electrochemical biosensor was fabricated by using molybdenum electrodes. The binding affinities of the conjugated SWCNTs to galectin-3 were quantified using electrochemical sensitivity measurements based on the differences in resistance together with typical I-V characterization. The electrochemical sensitivity measurements of the d-(+)-galactose-conjugated SWCNTs differed significantly between the samples with and without galectin-3. This indicates that d-(+)-galactose-conjugated SWCNTs are potentially useful electrochemical biosensors for the detection of cancer marker galectin-3.

## 1. Introduction

Galectins, a family of β-galactoside-binding proteins, have been strongly implicated in cancer and may be useful targets for the development of new cancer detection methods [1,2]. So far, 14 mammalian galectins have been identified, all of which contain a conserved carbohydrate recognition binding domain (CRD) of approximately 130 amino acids. Of the galectins, galectin-3 is the most studied member of the galectin family. High levels of circulating galectin-3 are correlated with an increased potential for malignancy in several types of cancer [3]. Currently, a convenient and economical method is not available for detecting galectins in tissue samples, although antibody based methods such as enzyme-linked immunosorbent assays (ELISA) and Western blotting are in use. A method based on a chemical probe shows potential as an alternative technique [4].

The development of advanced biosensor devices has emerged as the most promising short-term application of carbon nanotubes (CNTs) in biology and medicine. CNTs offer new opportunities for rapid, sensitive, and label-free detection of biological agents, and biofunctionalization confers selectivity of detection on the CNTs [5]. The use of single-walled CNTs (SWCNTs) in biosensors has been reported [6–18]. Currently, research on CNT-based biosensors is focused on exploiting the development of CNT electrodes for the electrochemical detection of biological agents, such as glucose [19], immunoglobulin G (IgG) [20], immunoglobulin E (IgE) [21,22], thrombin [23], and total prostate-specific antigen (T-PSA) as a cancer marker [24]. Several research groups have explored the electrochemical detection of biological molecules with electrodes consisting of CNTs in their pristine or modified forms [19,20–27]. Since their introduction into electrochemistry, CNT electrodes have demonstrated enhanced sensitivity compared to conventional carbon electrode [6,20–24]. In addition, electronic changes in the behavior of SWCNTs have been detected when they interact with small biological molecules and proteins [14,19,23,24,27–29]. A field-effect transistor (FET), composed of an individual pristine SWCNT, changes resistance upon exposure to proteins [14].

It is important to develop convenient and inexpensive methods for detecting and quantifying multiple galectins in tissues, both for biological studies and for future diagnosis using clinical samples [4]. While the previous methods, such as ELISA and Western blotting, are useful in research, they may not be practical for the routine analysis of clinical samples, and most are limited to the detection of a single galectin [4]. Most current efforts to develop alternatives have been based on selective galectin labeling using chemical probes. In our experiments, we used SWCNTs as chemical probes. To our knowledge, this is the first time that d-(+)-galactose-conjugated SWCNTs have been used as chemical probes to detect galectin-3.

Based on our previous finding [30] that d-(+)-galactose at a concentration of 0.5–1 μg/100 μL can bind to galectin-3 without structural changes, we investigated the binding affinity of galectin-3 at a nanoscale in electrochemical detection studies using a d-(+)-galactose-conjugated CNTs biosensor. Here, we determined the binding affinity of d-(+)-galactose-conjugated CNTs for the detection of the cancer marker galectin-3. This study is intended to provide preliminary information on the potential of d-(+)-galactose-conjugated CNTs as efficient nanobiosensors for the detection of the cancer marker galectin-3.

## 2. Results and Discussion

### 2.1. Binding Affinities of d-(+)-Galactose for Galectins

From our preliminary studies of the binding of d-(+)-galactose to galectins at various concentrations (0.25–2 μg/100 μL), we found that the absorbance intensity at 405 nm for the binding was approximately 0.5 for galectin-3 and 0.9 for galectin-8. In addition, the fraction of galectins bound to d-(+)-galactose increased during up to 30 min of incubation time. Moreover, no structural damage to galectin-3 or galectin-8 occurred in the binding studies, and the galectins retained their activity [30].

### 2.2. Purification and Functionalization of SWCNTs

As a first step in the biofunctionalization of SWCNTs, they were purified to remove amorphous carbon and transition metal impurities. In particular, the SWCNTs were purified to remove transition metals, such as Fe, Ni, and Co, as these impurities may result in reduction-oxidation reactions during the manufacture of biosensors using electrochemical methods. After purification, a dispersion step was used to scatter the bundles of SWCNTs for the functionalization step.

The results of physical functionalization indicated that there was no π stacking or mutual bonding forces between d-(+)-galactose and SWCNTs. However, the introduction of chemical functional groups, such as –COOH and –COCl, did not cause any major structural alterations, as confirmed using field emission-scanning electron microscopy (FE-SEM; JEOL 6700F, JEOL, Tokyo, Japan), high-resolution transmission electron microscopy (HR-TEM; JEOL 2010F; JEOL), Fourier transform-Raman spectroscopy (FT-Raman; RM 1,000-Invia, Reinshaw, Gloucestershire, UK), and x-ray photoelectron spectroscopy (XPS; PHI 5100, Physical Electronics, Chanhassen, MN, USA). From the XPS Cl_2P_ analysis, no Cl-related peak was observed after treatment with HNO_3_/NaClO_3_, although a peak was observed after subsequent SOCl_2_ treatment. This latter observation confirmed the chemical functionalization on the surface of the SWCNTs. By contrast, significant structural alterations were observed in the FT-Raman analysis after using fluorination as another chemical functionalization process of SWCNTs. A coating of amorphous carbons on the surface of the fluorinated SWCNTs was observed using FE-SEM and HR-TEM, suggesting that excess fluorine bound to the surface of SWCNTs, which unzipped the SWCNT structure. This confirmed that fluorinated d-(+)-galactose-immobilized SWCNTs are unsuitable for use in the manufacture of electrochemical biosensors.

### 2.3. Immobilization of d-(+)-Galactose on SWCNTs

**Figure 1.**
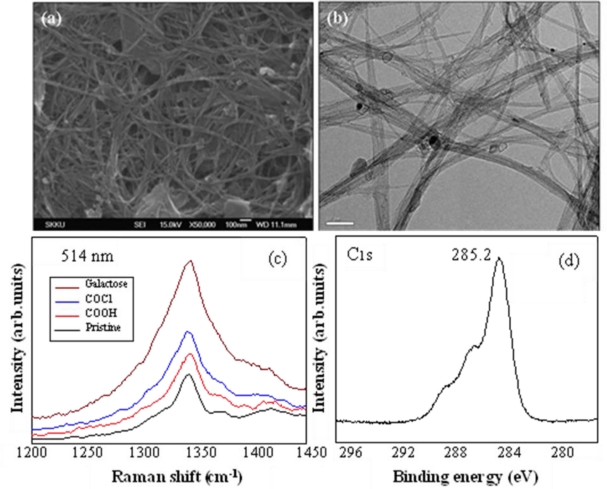
Images and analysis of SWCNTS after introducing d-(+)-galactose. **(a)** FE-SEM image, scale bar 100 nm; **(b)** HR-TEM image, scale bar 0.5 μm; **(c)** d-band in FT-Raman; **(d)** XPS C_1S_.

The FE-SEM and HR-TEM observations showed that the structure of the SWCNTs did not change after the reaction with d-(+)-galactose on SWCNTs functionalized with –COCl (Figure 1a and 1b). As shown in Figure 1c, the d-band related to sp^3^ was markedly increased due to sp^3^ bonding of d-(+)-galactose immobilized on the surface of the SWCNTs. After the reaction with d-(+)-galactose, the peak of sp^3^ bonding was increased markedly at 285.2 eV in the XPS C_1S_ analysis (Figure 1d).

**Figure 2.**
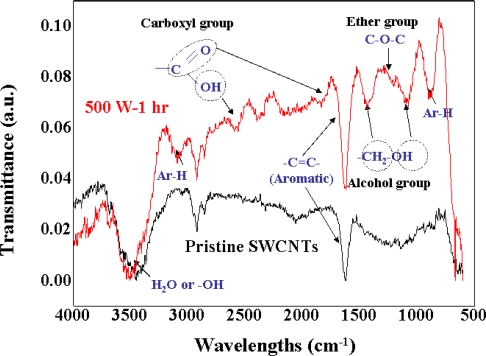
FT-IR analysis after introducing d-(+)-galactose on the surface of SWCNT-COCl. The stretching frequencies of ether and alcoholic linkages (C–O–C, –CH_2_–O) were seen around 1000∼1300 cm^−1^, the carbonyl of carboxylic acid at ∼1700 cm^−1^, and the –OH of carboxylic acid at ∼2500 and ∼3500 cm^−1^.

In addition, the hydroxyl group, hydrogen, and alcohol group of d-(+)-galactose that appeared after the introduction of d-(+)-galactose were observed using Fourier transform infrared spectroscopy (FT-IR; Bruker Optics, Billerica, MA, USA) (Figure 2). From these observations, we confirmed that the chemically functionalized SWCNTs were safely immobilized with d-(+)-galactose without undergoing any structural alterations. Finally, the SWCNTs immobilized with d-(+)-galactose were dispersed as individual SWCNTs in dichloroethane (DCE) and this observation was confirmed using atomic force microscopy (AFM; SPA-400, Seiko Instruments, Chiba-shi, Japan).

### 2.4. Electrochemical Biosensor Using SWCNTs Immobilized with d-(+)-Galactose

**Figure 3.**
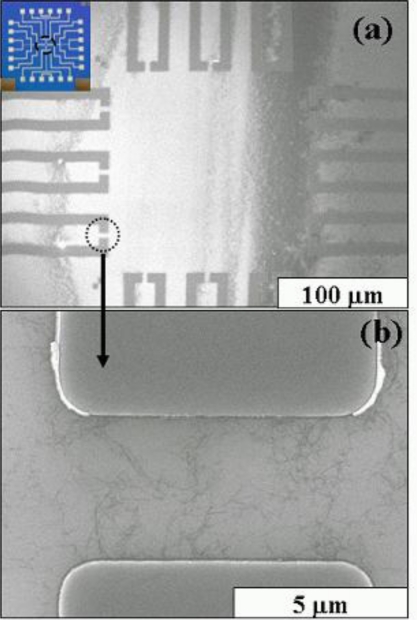
FE-SEM images of the molybdenum electrodes. **(a)** Twelve pairs of Mo Electrodes (inset: 1 × 1 cm area of the patterned electrode); **(b)** Dispersed SWCNTs between two Mo electrodes in the region outlined with the black dotted circle in **(**a**)**. The scale bars in **(**a**)** and **(**b**)** are 100 μm and 0.5 μm, respectively.

Individually dispersed SWCNTs were used to prepare electrochemical biosensors using d-(+)-galactose-immobilized SWCNTs. Well-dispersed SWCNTs in DCE were dropped onto the surface of a SiO_2_ substrate to fabricate molybdenum (Mo) electrodes as shown in Figure 3, and then used to measure current-voltage (I–V) characteristics and sensitivity based on differences in the resistance of samples in the presence or absence of galectin-3, without using other reference electrodes. Here, the primary effect of the binding of galectin-3 with SWCNTs is a charge-transfer reaction that involves the donation of electrons from galectin-3 to SWCNTs. The SWCNT-FET device may also be sufficiently sensitive to detect galectin-3 at low target concentrations. In these ways, the detection of galectin-3 is based not on measuring the oxidation-reduction potential of galectin-3 but on the I–V difference that occurs on the surface of SWCNTs when galectin-3 is bound to d-(+)-galactose-conjugated SWCNTs. Thus, despite a small electrochemical window, we used Mo electrodes without other reference electrodes to reduce dark current in SWCNT-FET and to increase the probability of p-type semiconduction during fabrication of an SWCNT-FET, and finally obtained high-sensitivity electrochemical biosensors. An electrochemical biosensor with a single strand of SWCNTs did not show sufficient sensitivity using Mo electrodes; it proved necessary to orient several dozen SWCNT strands between two Mo electrodes to function as a d-(+)-galactose-conjugated SWCNT biosensor.

**Figure 4.**
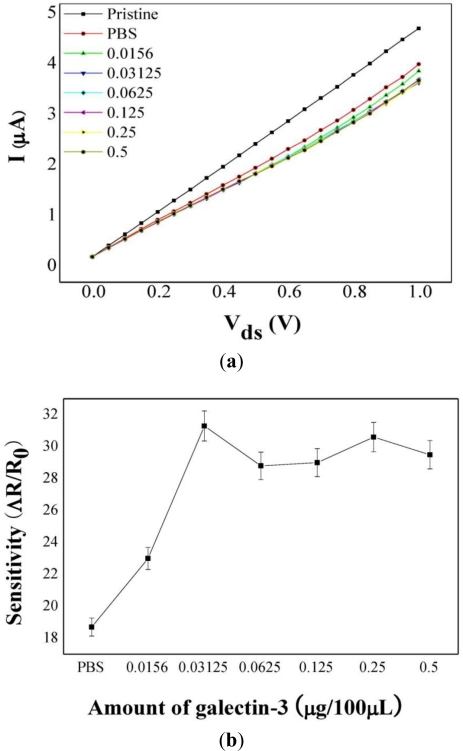
Typical graphical data for **(a)** I-V measurements and **(b)** the sensitivities of samples with or without galectin-3. The typical I-V characteristics were measured using the prepared Mo electrodes. SWCNTs dispersed between the Mo electrodes were exposed to various concentrations of galectin-3 in PBS (two-fold serial dilutions from 0.5 to 0.0156 μg/100 μL). The results were obtained from three different experiments. Each bar represents the mean standard deviation.

The sensitivity of a biosensor using SWCNTs dispersed between two Mo electrodes was defined as the difference in the resistance between sample solutions with or without galectin-3 and blank controls. The sensitivity was calculated using the formula [(Rm − R_0_)/R_0_], where Rm and R_0_ are the resistance of the samples with or without galectin-3 and that of the blank controls, respectively. Typical I-V measurements and sensitivities using the samples with or without galectin-3 are shown in Figure 4; the currents decreased after galectin-3 was bound to the d-(+)-galactose-conjugated SWCNTs based on the p-type characteristics of SWCNT-FETs [14,31]. Taking into account the fact that the SWCNTs exhibited p-type electronic behavior before adsorption of galectin-3, presumably due to doping from environmental oxygen, the current change after exposure to galectin-3 indicates an electron transfer from galectin-3 to the SWCNTs. A hybrid of galectin-3 and SWCNTs has been shown to be sensitive to the presence of d-(+)-galactose, which specifically binds to galectin-3, resulting in a decreased current. In this study, the sensitivity of the sample without galectin-3 was 18.7, whereas for samples with galectin-3, the maximum sensitivity was 31.3 (0.3125 μg/100 μL). The sensitivity of the samples increased with the galectin-3 concentration over a limited concentration range (0.0156–0.03125 μg/100 μL) (Figure 4b). From these results, we found that the resistance increased with the concentration of galectin-3 bound to the d-(+)-galactose-conjugated SWCNTs, and that the resistance was not further increased with concentration of galectin-3 higher than 0.03125 μg/100 μL because of the saturation of the majority carriers on the SWCNTs by the action of galectin-3 as an electron donor [14,31]. Finally, we confirmed that the sensitivity for the samples with galectin-3 increased with the concentration of galectin-3 in the binding reaction over a limited concentration range. These observations imply that d-(+)-galactose-conjugated SWCNTs are potentially useful as electrochemical nanobiosensors for the cancer marker galectin-3.

Galectin-3 is a non-immunoglobulin in nature and capable of specific recognition of and reversible binding to carbohydrate moieties of d-(+)-galactose-conjugated SWCNTs without altering the covalent structure of any of the recognized glycosyl ligands. In this study, SWCNT-FETs provided a simple detection mechanism between galectin-3 and d-(+)-galactose-conjugated SWCNTs without using reference electrodes. In addition, they resulted in a specific detection method with not only high selectivity based on the lectin characteristics of galectin-3 but also high sensitivity in the nanoscale range, based on a much larger diameter of galectin-3 compared with that of SWCNT channels (1–2 nm). Another advantage is that the binding between galectin-3 and d-(+)-galactose-conjugated SWCNTs is less reversible and more stable compared with non-covalent bonds in nature, such as antigen-antibody reactions based on non-covalent bonds including hydrogen bonds, electrostatic bonds, Van der Waals forces, and hydrophobic bonds. Therefore, the SWCNT-FET in this study offers several advantages, as mentioned above. Such an electrochemical sensor is small and rapid enough to detect the cancer marker galectin-3. In addition, the electrochemical sensor is extremely sensitive; all the current passes through the detection point. Most importantly, at a later stage, the SWCNTs-FET could be made specific to individual molecules; their response to different species could potentially be varied in a controlled way using chemical and biological functionalization.

In conclusion, this investigation of the binding sensitivity based on the resistance difference confirmed that d-(+)-galactose-conjugated SWCNTs are very efficient detection tools for selective binding to galectin-3, a biomarker on the surface of cancer cells. As the detection level demonstrated was within the nanoscale range (0.0156–0.03125 μg/100 μL), the SWCNT conjugates developed in this study could be used as efficient nanobiosensors for galectin-3. Furthermore, a study of the binding sensitivity of d-(+)-galactose-conjugated SWCNTs for the cancer marker galectin-3 would provide comprehensive information pertinent to their potential for further development as nanobiosensors. To use SWCNT conjugates for cancer detection, it must be possible to measure galectin-3 reproducibly using a reliable and widely available assay. Further studies using SWCNT conjugates as new chemical probes are required to develop reliable methods for detecting galectin-3 in routine clinical samples.

## 3. Experimental Section

### 3.1. Reagents

Galectins were purchased from PeproTech Asia (Rehovot, Israel) and R & D Systems (Minneapolis, MN, USA). Antibodies were purchased from Chemicon (Temecula, CA, USA) and Santa Cruz Biotechnology (Santa Cruz, CA, USA). β-d-(+)-Galactose and other chemical reagents were purchased from Sigma-Aldrich and used without further purification. Single-walled carbon nanotubes synthesized by the arc charge method were purchased from Iljin Nanotech (Seoul, Korea). All solutions were prepared using deionized water (Millipore, Bedford, MA, USA). The phosphate buffered saline (PBS) solutions adjusted to pH 7.4 and pH 7.2 as a supporting electrolyte were purchased from Invitrogen (Carlsbad, CA, USA).

### 3.2. Instrumentation

All experiments including purification, functionalization, immobilization, and dispersion steps, and electrochemical studies were performed using the research facilities at the Center for Nanotubes and Nanostructured Composites (CNNC) of Sungkyunkwan University (Suwon, Korea), including FE-SEM, HR-TEM, FT-Raman spectroscopy, XPS analysis, FT-IR, and AFM.

### 3.3. Preliminary Tests of Binding Affinity of d-(+)-Galactose and Galectin-3

Galectin-3 solutions were prepared at initial concentrations of 0.25–2 μg/100 μL in PBS (pH 7.4). All experiments were performed in the research facilities of the Biohealth Products Research Center (BPRC) of Inje University (Gimhae, Korea). In our preliminary test, we used ELISA, sodium dodecyl sulfate polyacrylamide gel electrophoresis (SDS-PAGE), and Bradford assays to examine the binding of galectins to d-(+)-galactose-conjugated carbon nanotubes [30].

### 3.4. Purification and Functionalization of SWCNTs

The SWCNTs including amorphous carbon particles as impurities were purified by heat treatment at 470 °C for 50 min under an air atmosphere. Then, transition metals used as catalysts in the process of SWCNT synthesis, such as Fe, Ni, and Co, were removed to inhibit the oxidation and reduction reactions in the process of biosensor manufacturing by stirring in aqueous HCl for 24 h.

Physical functionalization was performed by treating the surface of SWCNTs with d-(+)-galactose. SWCNTs were mixed with d-(+)-galactose at weight ratios of 1:30, 1:50, and 1:100. In addition, purified SWCNTs were prepared for chemical functionalization on their surfaces. Two different methods, *i.e.*, introduction of –COCl groups at the end of the CNTs and fluorination of CNTs, were used for chemical functionalization of SWCNTs. For introduction of –COCl groups, aliquots of 50 mg of purified SWCNTs were added to 100 mL of 70% HNO_3_. Then, 250 mg of NaClO_3_ was added to the resulting reactants under reflux at 100 °C, followed by rinsing with distilled water and alcohol, and poured into 100 mL of DMF solution. After full dispersal, 50 mL of SOCl_2_ were added to the resulting reactants under reflux at 100 °C. The introduction of –COOH and –COCl groups on the surface of SWCNTs was examined by FE-SEM, FT-Raman and XPS. For fluorination of CNTs, SWCNTs were introduced into the Ni reaction chamber and fluorinated by passage of 0.2 bar of F_2_ gas into the chamber at 25 °C for 30 min. The fluorination of SWCNTs was also examined by HR-TEM, FE-SEM, and FT-Raman.

### 3.5. Immobilization of d-(+)-Galactose on SWCNTs and Dispersion of SWCNTs Immobilized with d-(+)-Galactose

An excess of d-(+)-galactose was added to the SWCNTs functionalized with SOCl_2._ The reactants were subsequently kept at 280 °C under a nitrogen atmosphere for 12 h, and rinsed with distilled water and alcohols. The immobilization was observed using FE-SEM, HR-TEM, FT-Raman, XPS, and FT-IR. The SWCNTs immobilized with d-(+)-galactose were dispersed in DCE using ultrasonic treatment and centrifuged at 17,000 rpm for 30 min. The supernatant was separated and spin-coated on the surface of silicon substrates. The degree of dispersion was observed using AFM.

### 3.6. Biosensor Preparation and Electrochemical Analysis

The samples of 0.1 mg of SWCNTs immobilized with d-(+)-galactose were sonicated in 100 mL of DCE for 15 h. After centrifuging the sample at 27,460 g for 3.5 h, the supernatant was dropped on a prepatterned SiO_2_ substrate. The Mo electrode pattern was printed on the SiO_2_ substrate using conventional photolithography. Well-dispersed SWCNTs in DCE were dropped onto the patterned Mo electrodes. Each electrode sample with well-dispersed SWCNTs was dried in air overnight. The morphology of the dispersed SWCNTs between the two Mo electrodes was observed using FE-SEM. The density of SWCNTs was determined by controlling the amounts of CNTs in the solution. The typical I-V characteristics were measured using the prepared Mo electrodes. In order to measure the sensitivity of the biosensor, the SWCNTs dispersed between the Mo electrodes were exposed to various concentrations of galectin-3 in PBS (two-fold serial dilutions from 0.5 to 0.0156 μg/100 μL). The change in resistance was measured for samples with or without galectin-3. Controls were also measured using blank tests.

## 4. Conclusions

The binding affinities of d-(+)-galactose-conjugated SWCNTs for galectin-3 were determined using the electrochemical detection of resistance differences based on typical I-V characterization. Samples with galectin-3 showed greater sensitivity than those without galectin-3. In addition, the sensitivity of the samples with galectin-3 increased with the amount of galectin-3 in the binding reaction with the components at acceptable levels. The biofunctionally conjugated SWCNT showed electrochemical sensing levels in the nanoscale range for detecting the cancer marker galectin-3. Therefore, d-(+)-galactose-conjugated SWCNTs are potentially useful electrochemical nanobiosensors for the cancer marker galectin-3.
